# Depression and anxiety among Macau residents during the COVID-19 outbreak: A network analysis perspective

**DOI:** 10.3389/fpsyt.2023.1159542

**Published:** 2023-04-26

**Authors:** He-Li Sun, Pen Chen, Yuan Feng, Tong Leong Si, Mei Ieng Lam, Ka-In Lok, Ines Hang Iao Chow, Zhaohui Su, Teris Cheung, Yi-Lang Tang, Todd Jackson, Sha Sha, Yu-Tao Xiang

**Affiliations:** ^1^Unit of Psychiatry, Department of Public Health and Medicinal Administration, Institute of Translational Medicine, Faculty of Health Sciences, University of Macau, Taipa, Macau SAR,, China; ^2^Centre for Cognitive and Brain Sciences, University of Macau, Taipa, Macau SAR, China; ^3^The National Clinical Research Center for Mental Disorders and Beijing Key Laboratory of Mental Disorders, Beijing Anding Hospital and the Advanced Innovation Center for Human Brain Protection, Capital Medical University, Beijing, China; ^4^Kiang Wu Nursing College of Macau, Macau, Macau SAR, China; ^5^Faculty of Health Sciences and Sports, Macao Polytechnic University, Macau, Macau SAR, China; ^6^School of Public Health, Southeast University, Nanjing, China; ^7^School of Nursing, Hong Kong Polytechnic University, Kowloon, Hong Kong SAR, China; ^8^Department of Psychiatry and Behavioral Sciences, Emory University, Atlanta, GA, United States; ^9^Atlanta VA Medical Center, Atlanta, GA, United States; ^10^Department of Psychology, University of Macau, Macau, Macau SAR, China

**Keywords:** depression, anxiety, comorbidity, network analysis, Macau

## Abstract

**Background:**

The 2019 novel coronavirus disease (COVID-19) outbreak affected people’s lifestyles and increased their risk for depressive and anxiety symptoms (depression and anxiety, respectively hereafter). We assessed depression and anxiety in residents of Macau during “the 6.18 COVID-19 outbreak” period and explored inter-connections of different symptoms from the perspective of network analysis.

**Methods:**

In this cross-sectional study, 1,008 Macau residents completed an online survey comprising the nine-item Patient Health Questionnaire (PHQ-9) and seven-item Generalized Anxiety Disorder Scale (GAD-7) to measure depression and anxiety, respectively. Central and bridge symptoms of the depression-anxiety network model were evaluated based on Expected Influence (EI) statistics, while a bootstrap procedure was used to test the stability and accuracy of the network model.

**Results:**

Descriptive analyses indicated the prevalence of depression was 62.5% [95% confidence interval (CI) = 59.47–65.44%], the prevalence of anxiety was 50.2% [95%CI = 47.12–53.28%], and 45.1% [95%CI = 42.09–48.22%] of participants experienced comorbid depression and anxiety. “Nervousness-Uncontrollable worry” (GADC) (EI = 1.15), “Irritability” (GAD6) (EI = 1.03), and “Excessive worry” (GAD3) (EI = 1.02) were the most central symptoms, while “Irritability” (GAD6) (bridge EI = 0.43), “restlessness” (GAD5) (bridge EI = 0.35), and “Sad Mood” (PHQ2) (bridge EI = 0.30) were key bridge symptoms that emerged in the network model.

**Conclusion:**

Nearly half of residents in Macau experienced comorbid depression and anxiety during the 6.18 COVID-19 outbreak. Central and bridge symptoms identified in this network analysis are plausible, specific targets for treatment and prevention of comorbid depression and anxiety related to this outbreak.

## 1. Introduction

The Coronavirus Disease-2019 (COVID-19) pandemic has caused long-lasting negative effects on mental health (1–3). According to a report from the World Health Organization (WHO), the prevalence of depressive and anxiety disorders rose by 25% in the first year of the COVID-19 pandemic (4). Macau, as a tourist center that depends on visitor consumption as the basis of its social and economic life, has suffered devastating economic losses due to longstanding COVID-19-related travel restrictions and quarantine policies (5). Consequently, mental health problems have been widespread among local residents (6). For example, an online survey in the early stage of the pandemic indicated that 38.5% of Macau residents reported depression, while 28.8% reported anxiety (7), both of which were higher than the overall prevalence rates from a meta-analysis of depression and anxiety in China (26.9 and 21.8%, respectively) (8). To prevent virus transmission risk among local residents, Macau adopted strict public health measures (9, 10). However, such measures contributed to losses of economic prosperity, mobility, and social contact, all of which can compromise mental health (11).

Since late 2019, Macau has been hit by six waves of COVID-19; consequently, the economy has not yet recovered to its pre-pandemic levels (12), and many residents are at risk for poor mental health. Previous research has found that long-term exposure to mental health problems such as depression increases the likelihood of suicide (13) and major medical conditions (e.g., cardiovascular diseases) (14). Similarly, anxiety is associated with impairments in social and cognitive functioning (15). Compared to depression or anxiety alone, comorbid depression and anxiety are related to even more impairments in overall functioning (16, 17). Clinically, comorbidity of depression and anxiety is common; for example, one study found that approximately 85% of those who were depressed also had anxiety symptoms, while up to 90% of people with anxiety disorders also had concomitant depression (18). In light of these findings, consideration of comorbid depression and anxiety during COVID-19 outbreaks may provide guidance for policymakers and health professionals in the development of timely and effective interventions.

Historically, cutoff values based on total scores from standard questionnaires have been used to screen for depression and anxiety in epidemiological research (19). Although this approach is useful for generating overall prevalence estimates, it does not capture the nature or salient associations between individual symptoms. Unlike traditional research approaches, network analysis approaches can quantify relationships between individual depressive and anxiety symptoms (20). In network analysis theory, psychiatric syndromes are conceptualized as interacting clusters of symptoms that include nodes representing observed variables (e.g., depressive and anxiety symptoms) and edges representing strengths of relations between nodes (21). In network models, nodes with stronger connections are more tightly clustered and assumed to influence one another to a greater degree (21). Furthermore, not all nodes are equally important in network models; centrality indexes such as strength and expected influence (EI) are used to identify the most central nodes or symptoms in a network model. The most central nodes are assumed to influence surrounding nodes and have a more prominent role in the onset and/or maintenance of a psychiatric syndrome (22). Additionally, network analysis is useful for understanding comorbidity of psychiatric problems and can help to identify key bridge symptoms that trigger symptom contagion and risk for co-occurring syndromes (23). Presumably, targeting central symptoms may help to prevent or treat particular disorders (24) while targeting bridge symptoms in a network model may aid in reducing comorbidity (25).

Several network analysis studies have examined psychiatric symptoms and interactions among Macau residents during the COVID-19 pandemic. For example, in a network model of depression alone, the most important nodes were fatigue, motor impairments, and guilt (26). In research on a depression-anxiety-insomnia network model during an early stage of the pandemic, restlessness emerged as the most influential node (7). Despite such findings, to date, no network analysis studies have examined the network structure of comorbid depression and anxiety in a community-based sample of Macau residents during the pandemic. To redress this gap, we conducted a study immediately after June 18, 2022 (6.18) COVID-19 outbreak in Macau to assess the prevalence of depression, anxiety, and their comorbidity among Macau community-dwellers and clarify key inter-relationships between different depressive and anxiety symptoms in this group.

## 2. Methods

### 2.1. Participants and survey procedures

This study was conducted between July 26, 2022, and September 9, 2022, using a convenience sampling method immediately after the “the 6.18 COVID-19 outbreak” which lasted from 18 June to 1 Aug 2022 in Macau (27, 28). A questionnaire was used to collect data *via* the smartphone-based application, *“Questionnaire Star”*.^1^ Participants were invited to take this survey through advertisements on major social network platforms including Wechat, QQ, Facebook, and Instagram. To be eligible, participants had to be: (1) residents living in Macau during “the 6.18 COVID-19 outbreak”; (2) 18 years of age or older; (3) able to comprehend the survey objectives and contents. Electronic written informed consent was provided by all participants on a voluntary and confidential basis. The University of Macau Institutional Review Board (IRB) approved this study protocol.

### 2.2. Measures

The following socio-demographic data were collected: age, gender, marital status, living situation, level of education, and employment situation. In addition, pandemic-relevant information was recorded, including personal economic loss caused during the COVID-19 pandemic, worry about COVID-19 infection, and experiences of being quarantined during the COVID-19 pandemic.

The validated Chinese version of the nine-item Patient Health Questionnaire (PHQ-9) (29, 30), was used to assess the presence and severity of depression. PHQ-9 items included sad mood, sleep, fatigue, appetite, guilty feelings, concentration problems, motor problems, and suicide ideation. Each item was scored on a 4-point Likert scale from “0” (not at all) to “3” (almost every day), with higher total scores (range: 0–27), reflecting more severe depression. The PHQ-9 has acceptable reliability (e.g., Cronbach’s alpha = 0.91) and construct validity in Chinese samples (31). The validated Chinese version of the seven-item Generalized Anxiety Disorder scale (GAD-7) (32, 33) was used to measure anxiety. The GAD-7 items included nervousness, uncontrollable worry, excessive worry, trouble relaxing, restlessness, irritability, and feeling afraid. Each item was rated on a 4-point Likert scale from “0” (not at all) to “3” (almost every day); higher total GAD scores (range: 0–21) reflected more severe anxiety. The GAD-7 also has satisfactory reliability (e.g., Cronbach’s alpha = 0.89)and construct validity in Chinese samples (32).

### 2.3. Data analysis

#### 2.3.1. Descriptive statistics

Frequency analyses and sample mean calculations were performed to describe the nature of the study sample.

#### 2.3.2. Network node selection

In network theory, each symptom item is shown as a node, while edges represent independent relationships between nodes. However, if two nodes are highly redundant in a network model, the true structure of the model might be distorted (34). Hence, before constructing the network model, we applied the *goldbricker function* to identify redundant nodes in the depression and anxiety network model for Macau residents. Following a previous study (35), if two nodes were highly correlated with all other nodes in the network model (i.e., statistically different correlation of less than 25%), they were combined into a new node based on the mean value of two items.

#### 2.3.3. Network construction

Considering item scores in this network model were continuous variables, we used a Graphical Gaussian Model (GGM), though continuous variables should have a normal multivariate distribution with this approach (36, 37). The Shapiro–Wilk normality test indicated item scores were not normally distributed (*W* = 0.93, value of *p* <2.2e-16) so a transformation program was applied to normalize data before conducting the GGM network analysis (38). In the GGM model, partial polychromic correlations were used to represent edge estimates (39). However, in GGM a large number of parameters can generate redundant edges; this bias is controlled with the least absolute shrinkage and selection operator (LASSO) procedure (40). LASSO can shrink weak edges to zero, resulting in a parsimonious network that describes node covariance with as few edges as possible. Finally, we used the extended Bayesian Information Criterion (EBIC) to choose the optimal model from all candidate sparse network models with a default tuning parameter of 0.5 (41).

#### 2.3.4. Network centrality and bridge nodes

After obtaining the optimal network, the Fruchterman-Reingold algorithm was used to identify the layout of nodes (42). We used expected influence (EI) values because these are the most commonly used centrality statistic and can measure overall weights of model edges and provide connectivity information in networks with both positive and negative edges (43). Similarly, bridge expected influence (bridge EI) values were used to identify bridge nodes that linked depression and anxiety symptom communities (44). In addition, node predictability was calculated to assess how well a specific node could be predicted by neighbor nodes in the model (45).

#### 2.3.5. Network stability and accuracy

To evaluate stability in the order of central and bridge symptoms, a “*case-dropping subset bootstrap”* procedure was used (46). When the correlation stability coefficient (CS-coefficient) is larger than 0.5, the order can be regarded as having acceptable stability (41). We also assessed the accuracy of edge weights using a *“non-parametric bootstrap procedure”* (47). Furthermore, *“bootstrapped difference tests”* were applied to evaluate whether edge weights varied substantially from one other (48). All data analyses were performed using R program (Version 4.1.0) packages “qgraph,” “networktools,” “bootnet,” and “ggplot2” (36, 49–52).

## 3. Results

### 3.1. Demographic information

In total, 1,020 Macau residents were invited to participate in this study. Of these, 1,008 (739 women, 269 men) met all study entry criteria and completed the assessment (participation rate = 98.8%). Conversely, the other 12 residents either refused the invitation or did not complete the assessment. The average age of participants was 34.85 years [standard deviation (SD) = 11.5 years]. A majority of participants had a college or higher education (82.4%), and lived with others (92.8%), while less than half were married (46.7%). Regarding COVID-19-related queries, most participants reported feeling worried about being infected (60.7%), and around two-thirds acknowledged financial losses during the pandemic (63.2%) (Table 1). The prevalence of depression (PHQ-9 cutoff value of ≥5) was 62.5% [95% confidence interval (CI) = 59.47–65.44%], the prevalence of anxiety (GAD-7 cutoff value of ≥5) was 50.2% [95%CI = 47.12–53.28%], and the prevalence of comorbid depression and anxiety was 45.1% [95%CI = 42.09–48.22%] in the sample.

**Table 1:** Socio-demographic and clinical information of Macau residents (*n* = 1,008).

Variables	*N* (%)
Male gender	269 (26.7)
Married	471 (46.7)
Live with others	935 (92.8)
College school and above	831 (82.4)
Employed during the COVID-19 pandemic	687 (63.2)
Being quarantined during the COVID-19 pandemic	109 (10.8)
Worried about COVID-19 infection	
No worry	396 (39.3)
Worried	462 (45.8)
Very worried	150 (14.9)
Financial losses	
No or minimal	370 (36.7)
Fair	405 (40.2)
Very much	233 (23.1)
	Mean (SD)
Age	34.85 (11.5)
PHQ-9 total	7.32 (6.0)
GAD total	5.32 (5.1)

PHQ-9, the 9-item Patient Health Questionnaire; GAD-7, 7-item Generalized Anxiety Disorder Scale; SD, standard deviation.

### 3.2. Network structure

The goldbricker function analysis identified two pairs of redundant nodes: “Sleep” (PHQ3)—“Appetite” (PHQ5), and “Nervousness” (GAD1)—“Uncontrollable worry” (GAD2), both of which had 14.28% of correlations significantly different from all other nodes in the network. The two depressive symptoms were combined as a new symptom “Sleep-Appetite” (PHQC), while the two anxiety symptoms were combined as a “Nervousness-Uncontrollable worry” (GADC) node. There were 14 nodes in the final network model, including eight depression nodes and six anxiety nodes (see Table 1).

**Figure 1:**
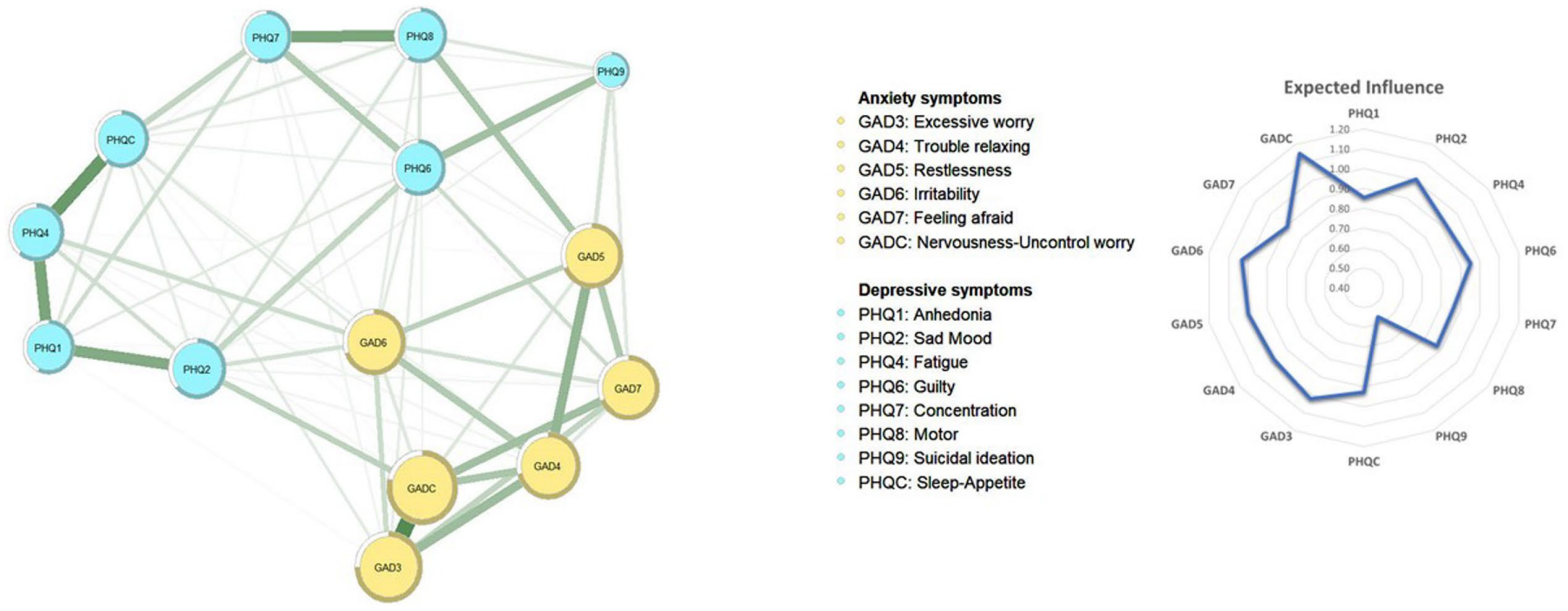
The network structure of depression and anxiety model in Macau residents.

**Figure 2:**
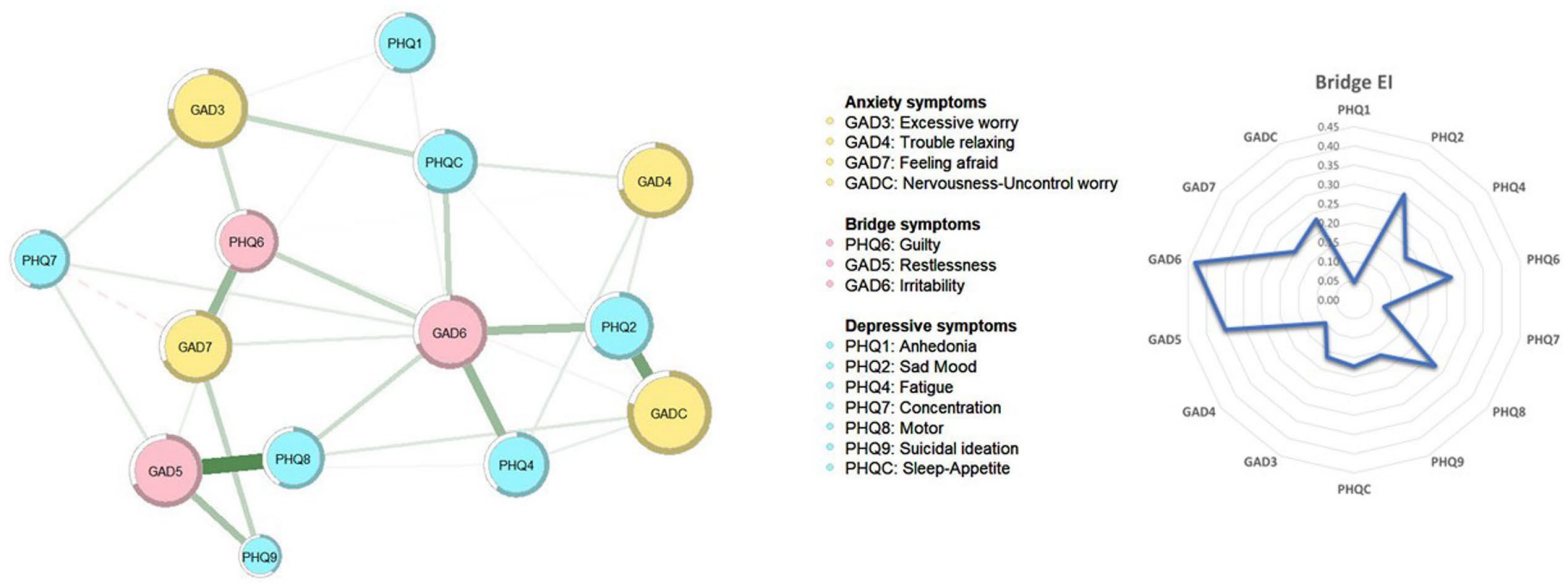
Bridge symptoms of depression and anxiety model in Macau residents.

For edges, the left panel of Figure 1 illustrates the overall network structure of depression and anxiety in the sample. In total, 14 nodes were retained in the network and 75.8% of the edges were set as non-zero, with a mean weight of 0.072. The strongest edge was between “Excessive worry” (GAD3) and “Nervousness-Uncontrollable worry” (GADC) in the anxiety community, followed by the edges between “Fatigue” (PHQ4) and “Sleep-Appetite” (PHQC), and between “Anhedonia” (PHQ-1) and “Fatigue” (PHQ4) in the depression community, all of which were significantly larger than the remaining edges (Supplementary Figure S1). Additionally, as shown in the left panel of Figure 2, the strongest edge linking two communities was between “Motor problems” (PHQ8) and “restlessness” (GAD-5), followed by “Sad Mood” (PHQ2) and “Nervousness-Uncontrollable worry” (GADC). Notably, majorities of edges within each community (edge weight range 0.008–0.292) were stronger than the edges across the two communities (edge weight range 0.001–0.186); the connection between “Restlessness” (GAD5) and “Motor problems” (PHQ8) was the strongest edge across symptom communities (edge weight: 0.186).

### 3.3. Network central and bridge symptoms

In terms of nodes, the right panel of Figure 1 presents EI values in the depression and anxiety network model. “Nervousness-Uncontrollable worry” (GADC) (EI = 1.15) was the most central symptom, followed by “Irritability” (GAD6) (EI = 1.03) and “Excessive worry” (GAD3) (EI = 1.02). Table 2 presents EI values for all symptoms. The right panel of Figure 2 shows the distribution of bridge EI connecting depression and anxiety communities. “Irritability” (GAD6) (bridge EI = 0.43) was the most important bridge symptom, followed by “restlessness” (GAD5) (bridge EI = 0.35) and “Sad Mood” (PHQ2) (bridge EI = 0.30). The node predictability analysis (Table 2) had a mean predictability of 0.632; Nervousness-Uncontrollable worry” (GADC) had the highest predictability (0.787).

**Table 2:** Descriptive statistics of depressive and anxiety symptoms in Macau residents.

Item abbreviation	Item content	Mean (SD)	Expected influence*	Predictability
PHQ1	Anhedonia	1.11 (0.928)	0.85	0.571
PHQ2	Sad Mood	0.93 (0.839)	1.01	0.637
PHQ4	Fatigue	1.25 (0.933)	0.92	0.612
PHQ6	Guilty	0.63 (0.915)	0.95	0.592
PHQ7	Concentration	0.68 (0.891)	0.86	0.557
PHQ8	Motor	0.49 (0.079)	0.87	0.583
PHQ9	Suicidal ideation	0.21 (0.594)	0.56	0.401
PHQC	Sleep – Appetite	1.00 (0.859)	0.93	0.604
GAD3	Excessive worry	0.85 (0.845)	1.02	0.749
GAD4	Trouble relaxing	0.84 (0.902)	0.98	0.708
GAD5	Restlessness	0.52 (0.787)	1.00	0.684
GAD6	Irritability	0.87 (0.890)	1.03	0.693
GAD7	Feeling afraid	0.71 (0.880)	0.89	0.682
GADC	Nervousness – Uncontrollable worry	0.77 (0.769)	1.15	0.787

*The values of were raw data generated from the network. PHQ-9: the 9-item Patient Health Questionnaire; GAD-7: 7-item Generalized Anxiety Disorder Scale; SD standard deviation.

### 3.4. Network stability

**Figure 3:**
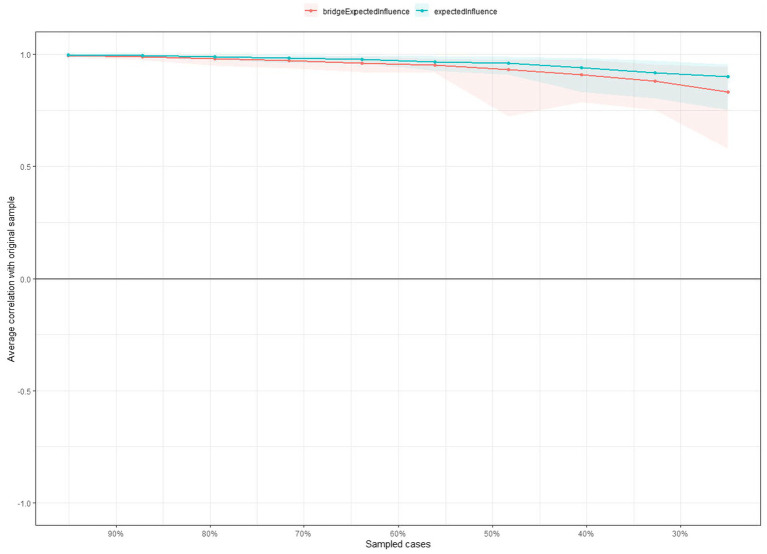
Centrality and bridge centrality indices tests.

Figure 3 shows the node stability of the network; both EI and bridge EI had a acceptable stability. The EI value of 0.75 indicated that even when 75% of the participants were dropped, results would not have a significant change compared with the original results. Supplementary Figure S2 presents confidence intervals, indicating that important EI and bridge EI were significantly larger than other nodes. Supplementary Figures S3, S4 illustrate edges and EI difference tests for the depression and anxiety model. Majorities of edges and nodes were stable and could be distinguished from each other.

## 4. Discussion

This study found that the prevalence of depression and anxiety comorbidity was high among Macau residents during “the 6.18 COVID-19 outbreak.” In the comorbidity network model, the most central symptoms were from the anxiety community and underscored how anxiety may have a critical role in activating the co-occurrence of depression and anxiety in the context of a local COVID-19 outbreak. However, this finding is also consistent with previous findings from a depression and anxiety comorbidity evolution study in which anxiety symptoms emerged earlier than depression (53). In addition, most edges within each community were stronger than edges linking the two communities, indicating that core symptoms within depression and anxiety communities were more closely entwined than links between symptoms across the two communities (54).

This study provided novel data related to depression and anxiety comorbidity in the context of pandemics, as both (i) “sleep” and “appetite,” and (ii) “nervousness” and “uncontrollable worry” were highly correlated symptom dyads. Comorbid depression and anxiety is common (55, 56), and the two syndromes exhibit affective, cognitive, psychological, and somatic modalities (57). However, only a few studies on depression-anxiety network models have examined symptom overlaps before constructed network maps (58); notably, these studies did not find overlaps within two syndromes in contrast to our findings. The discrepancy between studies may be partly due to differences in research methodology, study samples, and settings (59). Furthermore, the findings of network analyses may differ as a function of study context (e.g., assessment under pandemic versus non-pandemic conditions) (60).

Our analyses showed that “sleep” and “appetite” reflected similar response tendencies in Macau residents (61) in the context of a local COVID-19 outbreak. Sleep changes and appetite variations are sensitive biological rhythms in depression (62) and sleep alterations could influence appetite and food reward (63). When normal life rhythms are punctuated by a sudden public health emergency (e.g., the COVID-19 outbreak), people typically had to remain at home, possibly leading to significant disruptions in sleep–wake routines and eating habits (64). In addition, GAD symptom items, “nervousness” and “uncontrollable worry” were highly redundant in the network model in line with the high correlation between these symptoms in a previous network analysis conducted on Chinese college students during a COVID-19 outbreak (58). In response to the 6.18 COVID-19 outbreak, the Macau government implemented a policy in which residents were required to stay at home except for emergency situations and mandatory COVID-19 testing (9). Compared with the four previous outbreaks in Macau, the number of COVID-19 cases rapidly increased from 83 to 791 cases within days (65). Consequently, residents were more likely to feel nervous, fearful about the infection and distressed by strict externally imposed quarantine measures that contributed to uncontrollable worries about the outbreak.

Identifying the most influential nodes in a network model is important for generating empirically grounded hypotheses regarding symptoms that are critical to the occurrence and development of comorbid disorders/syndromes (43). In this network model, “Nervousness-Uncontrollable worry” (GADC) and “Excessive worry” (GAD3) were the most central nodes, a finding that may reflect threat-related symptoms of dysphoria that are most salient in the context of a sudden community outbreak of COVID-19 and associated disruptions to daily life routines (64). Furthermore, while constant local COVID-19-related news coverage in mass media during the outbreak may have provided important information about remaining safe, such exposure may have also perpetuated nervousness and anxiety throughout a community expected to remain on “red alert” over an extended period of time (66). Additionally, worry may have been perpetuated by uncertainty regarding risk and responses to infection, the timeline of returns to normal routines, the status of one’s employment, and financial stability. For example, in our survey, nearly two-thirds of participants acknowledged experiencing financial losses during the outbreak. Even if a society resumes normal routines, personal and societal economic difficulties can linger (67) and contribute to ongoing worry.

“Irritability” (GAD6) was also among the most important central symptoms and bridge symptoms in the network model; this finding implies that reducing irritability should be useful in reducing anxiety as well as the co-occurrence of depression and anxiety in the context of ongoing community emergency situations. Irritability reflects heightened arousal that often arises from blocked goal attainment and is a typical feature of anger (68). During the 6.18 COVID-19 outbreak, most people could not leave their residences freely (69) so both physical movement and autonomy were significantly restricted. In the context of such restrictions, increased anger and irritability are understandable emotional reactions. Exacerbating such limitations, Macau is the most densely populated area in the world (70) and most residents live in high-rise apartment buildings; restrictions to mobility and autonomy in this context could increase stress reactions and irritability (71).

Finally, “Restlessness” (GAD5) and “Sad mood” (PHQ2) emerged as key bridge symptoms in this study in line with recent findings from another depression-anxiety network analysis (72). Deactivation treatment for bridge symptoms has clinical promise (43). Based on our findings, treating “Restlessness” in people who experience elevations in anxiety may have colateral benefits in reducing current depressive symptomatology or risk for developing depression. For instance, meditation and mindfulness exercises, regular physical exercise, and social support from significant others in one’s social network may all aid in reducing restlessness (43). Similarly, treating “Sad mood” in depression may extend to relieving or preventing anxiety symptoms consistent with experimental evidence indicating induced sadness can increase anxiety responses (73).

Despite its potential implications, the main limitations of this study should be mentioned. First, this was a cross-sectional study so causal directions between symptoms could not be determined. Prospective studies and randomized control trials targetting comorbidity may help to clarify causal directions in future studies. Second, due to restrictions and human resource demands related to the outbreak, in-person structured clinical interviews could not be conducted and depressive and anxiety symptoms were measured *via* validated questionnaires. Consequently, diagnoses of depression and anxiety disorders could not be made. Third, responses biases related to recall and/or social desirability could not be ruled out as influences due to the reliance on self-report measures of experience. Fourth, various factors associated with depressive and anxiety symptoms, including social support and stressful life events, were not assessed in an effort to maintain reasonable response burdens on unpaid volunteers. Finally, for logistical reasons, convenience sampling rather than random sampling was used, so representativeness of the study sample may have been reduced. On a related note, majorities of participants were women and young adults (mean age = 33.85), so findings may not generalize well to all age groups or across gender.

In conclusion, we found that anxiety and depression comorbidity was common among Macau residents during the 6.18 COVID-19 outbreak. “Nervousness-Uncontrollable worry” (GADC), “Irritability” (GAD6), and “Excessive worry” (GAD3) were the most central symptoms, while “Irritability” (GAD6), “restlessness” (GAD5), and “Sad Mood” (PHQ2) were the most important bridge symptoms in the depression-anxiety network model, suggesting that these symptoms would be useful targets for intervention within this context. For example, cognitive behavioral therapy that targets these symptoms based on behavioral activation and cognitive restructuring could be helpful for those who experience comorbid depression and anxiety during pandemic outbreaks.

## Data availability statement

The datasets presented in this article are not readily available because the University of Macau Institutional Review Board (IRB) that approved the study prohibits the authors from making publicly available the research dataset of clinical studies. Requests to access the datasets should be directed to xyutly@gmail.com.

## Ethics statement

The studies involving human participants were reviewed and approved by University of Macau Institutional Review Board (IRB). The patients/participants provided their written informed consent to participate in this study.

## Author contributions

YF, SS, and Y-TX: study design. PC, TS, ML, K-IL, IC, ZS, and TC: data collection, analysis, and interpretation. H-LS, Y-LT, and Y-TX: drafting of the manuscript. TJ: critical revision of the manuscript. All authors contributed to the article and approved the submitted version.

## Funding

This study was supported by the National Science and Technology Major Project for investigational new drug (2018ZX09201-014), the Beijing Hospitals Authority Clinical Medicine Development of special funding support (XMLX202128), and the University of Macau (MYRG2019-00066-FHS; MYRG2022-00187-FHS).

## Conflict of interest

The authors declare that the research was conducted in the absence of any commercial or financial relationships that could be construed as a potential conflict of interest.

The handling editor YZ declared a shared affiliation with the authors YF and SS at the time of review.

## Publisher’s note

All claims expressed in this article are solely those of the authors and do not necessarily represent those of their affiliated organizations, or those of the publisher, the editors and the reviewers. Any product that may be evaluated in this article, or claim that may be made by its manufacturer, is not guaranteed or endorsed by the publisher.

## Footnotes

^1^ http://www.wjx.cn
