# Supplementary material for: Depression and anxiety among Macau residents during the COVID-19 outbreak: A network analysis perspective
Source: Front Psychiatry. 2023 Apr 26;14:1159542. doi: 10.3389/fpsyt.2023.1159542 (PMC10169684; doi:10.3389/fpsyt.2023.1159542)
Supplement: Supplementary file 1 [file Data_Sheet_1.docx]

**Supplementary materials**

**Figure legends**

Figure S1. Bootstrapped confidence intervals of edge weights.

Figure S2. Bootstrapped test for confidence intervals of EI and bridge EI of depression and anxiety model

Figure S3. Bootstrapped difference test for edge weight of depression and anxiety model

Figure S4. Bootstrapped difference test for expected influence of depression and anxiety model

**Table S1. Weighted adjacency matrix of the** **depression and anxiety model**

|  | **PHQ1** | **PHQ2** | **PHQ4** | **PHQ6** | **PHQ7** | **PHQ8** | **PHQ9** | **PHQC** | **GAD3** | **GAD4** | **GAD5** | **GAD6** | **GAD7** | **GADC** |
| --- | --- | --- | --- | --- | --- | --- | --- | --- | --- | --- | --- | --- | --- | --- |
| **PHQ1** |  |  |  |  |  |  |  |  |  |  |  |  |  |  |
| **PHQ2** | 0.275 |  |  |  |  |  |  |  |  |  |  |  |  |  |
| **PHQ4** | 0.292 | 0.103 |  |  |  |  |  |  |  |  |  |  |  |  |
| **PHQ6** | 0.061 | 0.129 | 0.000 |  |  |  |  |  |  |  |  |  |  |  |
| **PHQ7** | 0.106 | 0.018 | 0.025 | 0.172 |  |  |  |  |  |  |  |  |  |  |
| **PHQ8** | 0.000 | 0.077 | 0.000 | 0.081 | 0.291 |  |  |  |  |  |  |  |  |  |
| **PHQ9** | 0.000 | 0.043 | 0.000 | 0.205 | 0.036 | 0.075 |  |  |  |  |  |  |  |  |
| **PHQC** | 0.074 | 0.057 | 0.328 | 0.041 | 0.134 | 0.073 | 0.045 |  |  |  |  |  |  |  |
| **GAD3** | 0.014 | 0.000 | 0.000 | 0.054 | 0.034 | 0.000 | 0.000 | 0.062 |  |  |  |  |  |  |
| **GAD4** | 0.000 | 0.030 | 0.029 | 0.003 | 0.000 | 0.000 | 0.000 | 0.036 | 0.217 |  |  |  |  |  |
| **GAD5** | 0.000 | 0.000 | 0.012 | 0.023 | 0.036 | 0.186 | 0.092 | 0.000 | 0.008 | 0.237 |  |  |  |  |
| **GAD6** | 0.017 | 0.098 | 0.109 | 0.061 | 0.028 | 0.054 | 0.000 | 0.064 | 0.113 | 0.170 | 0.149 |  |  |  |
| **GAD7** | 0.013 | 0.030 | 0.000 | 0.108 | -0.018 | 0.000 | 0.068 | 0.000 | 0.137 | 0.071 | 0.181 | 0.098 |  |  |
| **GADC** | 0.001 | 0.145 | 0.025 | 0.014 | 0.000 | 0.034 | 0.000 | 0.013 | 0.382 | 0.187 | 0.073 | 0.069 | 0.206 |  |

**
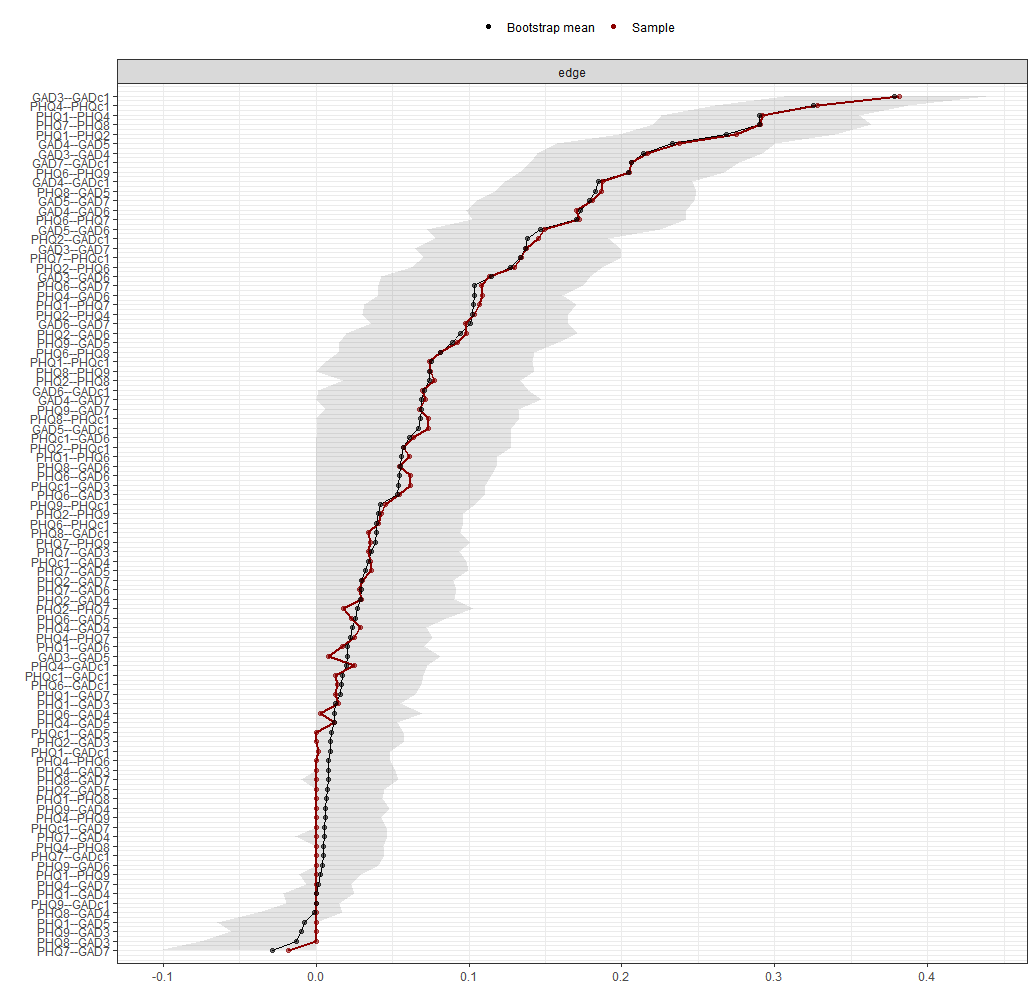
**

**Figure S1. Bootstrapped confidence intervals of edge weights**

**
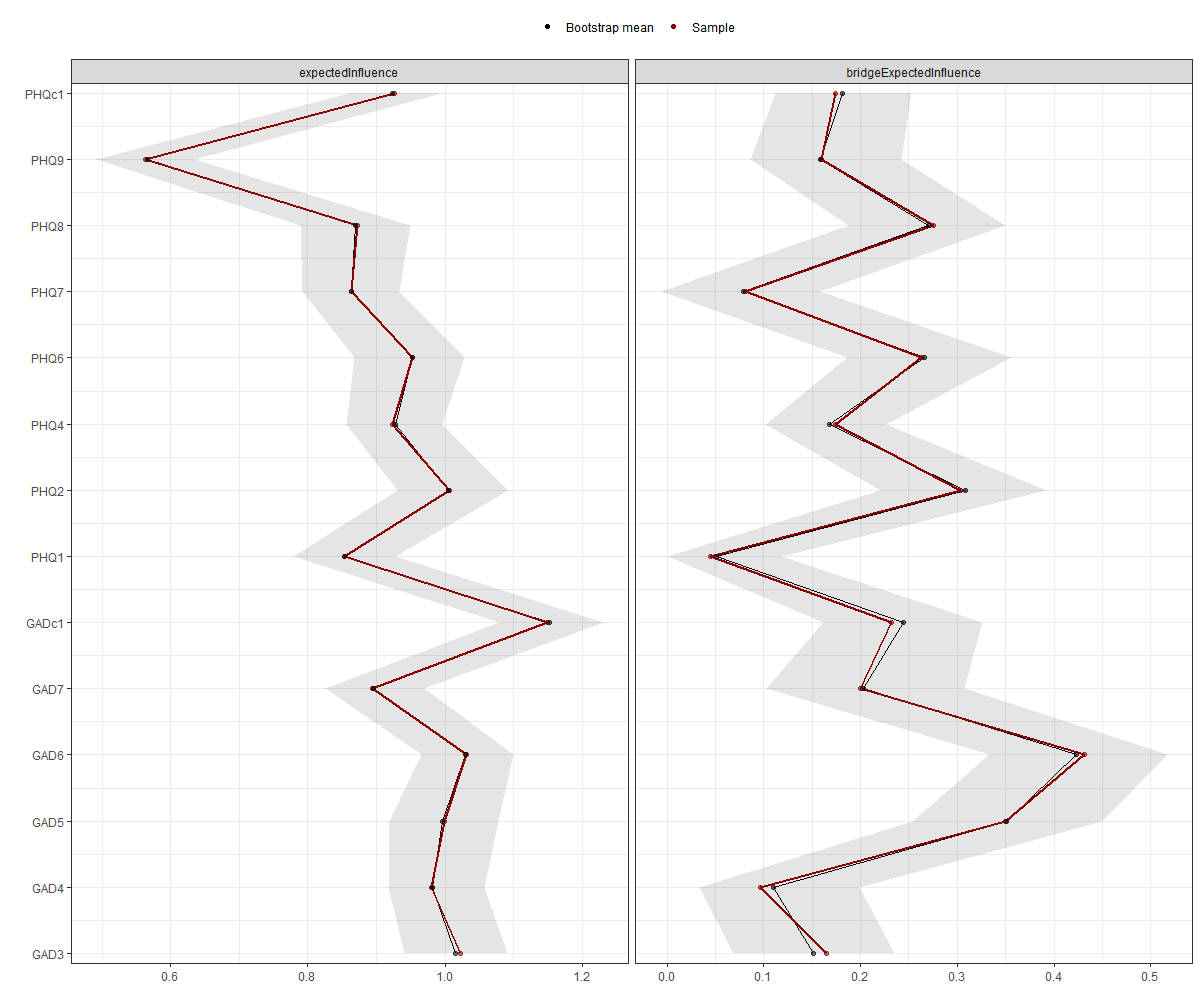
**

**Figure S2. Bootstrapped test for confidence intervals of EI and bridge EI of depression and anxiety model**

**
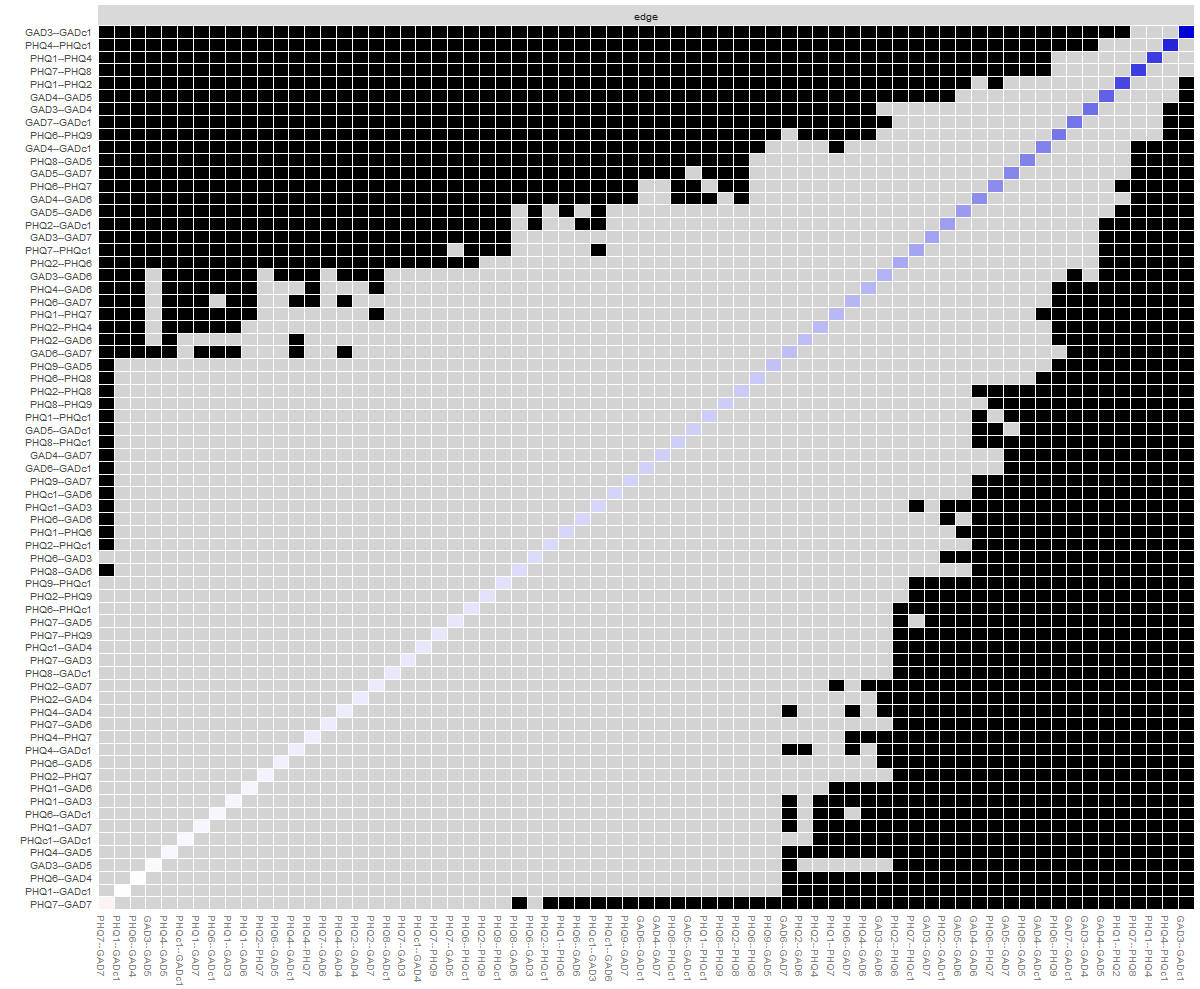
**

**Figure S3. Bootstrapped difference test for edge weight of depression and anxiety model**

**
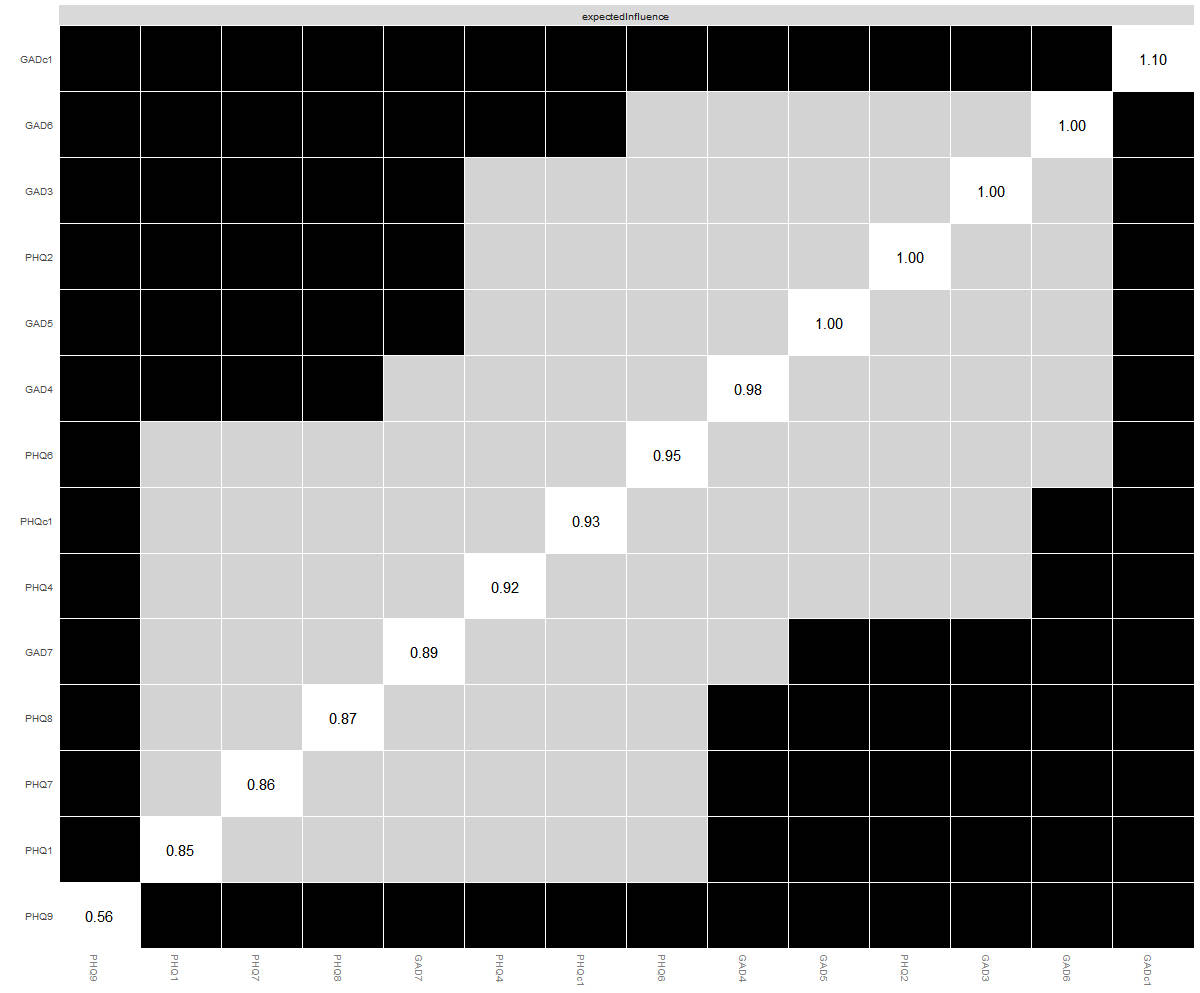
**

**Figure S4. Bootstrapped difference test for expected influence of depression and anxiety model**
